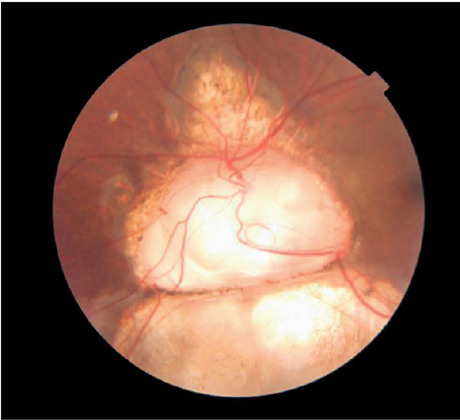# Optic disc coloboma - A hidden masquerader

**DOI:** 10.5935/0004-2749.2024-0105

**Published:** 2024-08-30

**Authors:** Ishan Tilak, Vyshakh V Kizhakkekara, Swathi Nagrajan, Nisha Chakkaravarthy

**Affiliations:** 1 Department of Ophthalmology, Mahatma Gandhi Medical College and Research Institute, Sri Balaji Vidyapeeth, Pillayarkuppam, Pondicherry India

Optic disc coloboma is a condition, present unilaterally or bilaterally, within the spectrum of optic nerve coloboma, characterized by a bowl-shaped excavation of the optic disc. The image depicts type 2 optic disc coloboma, as per Ida Mann’s classification, detected incidentally during routine ophthalmic screening in a patient with 20/20 vision^(1)^. Despite its asymptomatic presentation, it is important not to overlook optic nerve coloboma, as it may be associated with macular schisis or retinal detachment^(2)^.

**Figure:**